# Evolutionary rescue in populations of *Pseudomonas fluorescens* across an antibiotic gradient

**DOI:** 10.1111/eva.12046

**Published:** 2013-02-04

**Authors:** Johan Ramsayer, Oliver Kaltz, Michael E Hochberg

**Affiliations:** 1 Institute of Evolutionary Sciences, University of Montpellier 2Montpellier, France; 2 Santa Fe InstituteSanta Fe, NM, 87501, USA

**Keywords:** antibiotic, evolutionary rescue, pharmacology, *Pseudomonas fluorescens*, Resistance

## Abstract

Environmental change represents a major threat to species persistence. When change is rapid, a population's only means of persisting may be to evolve resistance. Understanding such ‘evolutionary rescues’ is important for conservation in the face of global change, but also in the agricultural and medical sciences, where the objective is rather population control or eradication. Theory predicts that evolutionary rescue is fostered by large populations and genetic variation, but this has yet to be tested. We replicated hundreds of populations of the bacterium *Pseudomonas fluorescens* SBW25 submitted to a range of doses of the antibiotic streptomycin. Consistent with theory, population size, and initial genetic diversity influenced population persistence and the evolution of antibiotic resistance. Although all treated populations suffered initial declines, those experiencing the smallest decreases were most likely to be evolutionarily rescued. Our results contribute to our understanding of how evolution may or may not save populations and species from extinction.

## Introduction

Rapid environmental change can cause population declines with undesired or desired effects. Global climate change, for instance, threatens biodiversity by contributing to observed population and species extinctions being one or more orders of magnitude above background rates (Dirzo and Raven 2003). Chemical applications, on the other hand, are an important means to control populations of pests, parasites and pathogens, and diseased cells and tissues, such as cancers (Lambert et al. 2011). Both of these contexts share the feature that a population submitted to a stress may either genetically adapt and grow before the population gets too small, or be unable to recover its losses and go extinct.

The theory of evolutionary rescue provides a conceptual framework linking demography and evolution in finite populations (Gomulkiewicz and Holt 1995; Holt et al. 2004; Orr and Unckless 2008). It thus combines the population dynamics occurring in declining populations with fundamental principles of population genetics, determining the fate of beneficial mutations. In the face of abrupt and lethal stress, adaptation may depend on the emergence of one or more mutations that promote individual survival and/or reproduction. These resistant types may already be present in the population, or may emerge during the decline following the onset of a stress. Characteristic of evolutionary rescue, population density may therefore exhibit a U-shaped curve (Bell and Gonzalez 2009), with the specific trajectory determined by the relative benefits of selected mutations and the time when they emerge (Orr and Unckless 2008). Theory predicts that whether or not natural selection can rescue a population from a stress will depend on the stress level, the initial population size (Gomulkiewicz and Holt 1995), the initial population genetic diversity, mutation rate, and costs and benefits of adaptive mutations (Willi et al. 2006; Kawecki 2008; Willi and Hoffmann 2009).

The ability to withstand and adapt to abiotic stress has been described for various organisms (McNeilly and Bradshaw 1968; Jasieniuk et al. 1996; Labbé et al. 2007), and often the physiological and genetic bases are well understood (Feder and Hofmann 1999; Sorensen et al. 2003). However, it is still largely unclear to what extent it is possible to quantify and predict the chance of evolutionary rescue in declining populations. Only very recently have experimental studies begun to use microbial systems to investigate evolutionary rescue, and follow trajectories in real time. These studies have examined the roles of population size (Bell and Gonzalez 2009) and migration and the rate of change in stress levels (Perron et al. 2008; Bell and Gonzalez 2011) in evolutionary rescues, but the drivers of rescue trajectories are largely unknown and no experimental study to our knowledge has demonstrated the possibility of predicting rescue based on initial population declines.

From a practical perspective, antibiotics are convenient stressors because their effects can be studied with small organisms (bacteria), thereby allowing investigation of rescue dynamics with sufficient replication under well-controlled conditions in the laboratory over relatively short time windows (hours to days). In addition, there are straightforward healthcare implications. Administered at sufficiently high doses, antibiotics rapidly kill sensitive bacteria unless they are phenotypically resistant, in such case, leading to treatment failure (Smith and Romesberg 2007). Clearly, understanding the factors that permit the undesired rescue of bacterial infections could be key to more general antibiotic therapy management strategies that aim at limiting the spread of resistance (Smith and Romesberg 2007; Baquero et al. 2011) and possible horizontal transfer of resistance genes to other bacterial species (Levin and Cornejo 2009).

Indeed, the evaluation of pharmacodynamics, that is, bacterial population dynamics under antibiotic stress, is part of standard protocols establishing antibiotic regimes for therapy (Regoes et al. 2004; Levin and Udekwu 2010). So-called ‘time-kill experiments’ track population growth along antibiotic dose gradients to establish minimal levels required to eradicate sensitive populations. These tests often show relatively rapid declines in mortality rate (Regoes et al. 2004), indicative of incipient rescue. However, these declines may not necessarily be caused by heritable resistance (Wiuff et al. 2005; Levin and Rozen 2006; Udekwu et al. 2009). Moreover, still, little is known about how ecological and epidemiological factors such as initial bacterial population size or standing genetic variation influence the emergence of heritable resistance.

Here, we tested theory on evolutionary rescue by submitting hundreds of populations of the Gram-negative bacterium *Pseudomonas fluorescens* SBW25, commonly used in experimental evolution studies (e.g., Rainey and Travisano 1998; Barrett et al. 2005; Venail et al. 2007), to different doses of the antibiotic streptomycin. Our pilot studies showed that some populations of *P. fluorescens* SBW25 persist after the addition of this antibiotic at concentrations lethal to the vast majority of individual bacterial cells. We varied both the initial population genetic diversity and initial population size to discover how these affect the probability of evolutionary rescue and the variability in outcomes in cases where populations are rescued. Consistent with theory, we found that a higher potential for genetic adaptation fostered the evolution of antibiotic resistance and evolutionary rescue. Although all treated populations suffered initial declines, when controlling for antibiotic dose, those experiencing the smallest initial decreases were the most likely to be rescued. Furthermore, larger populations that survived antibiotics produced more variable densities at the end of the experiment, suggesting different population trajectories, possibly due to different emergence times or different relative benefits of favorable mutations. Taken together, these results show how the population characteristics may influence evolutionary rescue.

## Materials and methods

### Experiment 1: Test for the effect of genetic diversity in evolutionary rescue

In this experiment, we prepared genetically diversified and clonal populations (see below) and exposed them to different doses of the antibiotic streptomycin (Sigma) for 53 h. We measured population sizes at different time points, postexposure. Two replicates of antibiotic-free controls were also grown for both diversified and clonal population treatments, making a total of 64 populations.

To prepare the diversified and clonal treatments, we initiated eight populations (each from a different, arbitrarily chosen clone) of *P. fluorescens* SBW25 in 30-mL glass vials, supplied with 6 mL of King's B medium (glycerol 10 mL/L; proteose peptone H3 20 g/L; K2HPO4 1.5 g/L; MgSO4 1.5 g/L; distilled water). Ten percent of each of these populations was serially transferred 14 times to a new microcosm with fresh KB medium every 2 or 3 days for a total of 30 days (approximately 100 bacterial generations) at 28°C under constant rotational agitation (200 rpm). On day 30, we pooled the eight populations into a single ‘master’ population, from which we initiated each population for the diversified treatment. Populations for the clonal treatments were obtained by isolating single clones from this master population on KB-agar plates. We verified that diversified populations had higher frequencies of resistant mutants than the nondiversified populations (see Supporting information, Assays 1 and 2 in Data S1; Fig. S1).

Experimental replicate populations were initiated by transferring 20 μL of the master population (genetically diversified treatments) or clonal populations (nondiversified treatments) in exponential growth phase into 30-mL glass vials, containing 2 mL of KB. Populations grew for 24 h at 28°C under constant agitation of 200 rpm. At 24 h postinoculation, 20 μL of each population was plated on KB-agar for subsequent counting of colony forming units (CFUs), and streptomycin was added to each vial at 0, 50, 100, or 200 μg/mL (for estimation of MIC_50_, see Supporting information, Assay 3 in Data S1).

Population sizes were measured by plating 20-μL samples (serially diluted between 10^−1^ and 10^−6^) and by counting CFUs at 4, 9, 22, 30, and 53 h post antibiotic introduction. Bacterial density for each vial was estimated by averaging counts from three different samples. Populations were considered ‘extinct’ when no CFUs were detected in the 53-h sample. The population size detection threshold was 333 bacterial cells in 2-mL vials (i.e., if we found only one CFU in only one of the three 20-μL samples diluted at 10^−1^).

Antibiotic resistance was assessed as follows. The KB-agar plates used for counting population sizes were maintained at 4°C to arrest bacterial growth. At the end of the experiment, one arbitrarily chosen clone of each population was reamplified in 2-mL KB microcosms for 24 h. Clones from surviving populations were taken from the last time step of the experiment, whereas clones from the extinct populations were taken from the last time step at which the population was still alive. Resistance to streptomycin was assayed by placing two drops (20 μL each) of vortexed culture onto KB-agar plates supplemented with either 50, 100, or 200 μg/mL of streptomycin just prior to the distribution of agar on plates. Each clone was tested against each of three streptomycin doses.

### Experiment 2: Test for the effect of population size on evolutionary rescue

In a second experiment, we investigated evolutionary rescue in two different volumes, the smaller being 200 μL of KB in 96-well plates, and the larger 1.5 mL in 24-well plates. We inoculated wells with 10% of final volumes from a population composed of five individual clones of stationary phase *P. fluorescens* SBW25 (after 24 h growth in KB). Treatment populations grew for 24 h at 28 °C under constant agitation (130 rpm). After 24 h, populations attained densities in the range of *c*. 2–5 × 10^9^ cells/mL. This indicates that populations were between 0.4 and 1 × 10^9^ cells in 0.2 mL wells, and between 3 and 7.5 × 10^9^ cells in 1.5-mL wells. On the basis of findings in experiment 1 and pilot experiments, we explored in more detail how populations were affected by lower streptomycin doses by exposing each population to one of five different concentrations of streptomycin (0, 30, 40, 50, 100 μg/mL). Each treatment was replicated 12 times, making a total of 120 populations. Moreover, based on the observation in experiment 1 that some of the rescued populations were still growing one day post antibiotic addition, we estimated total population sizes by plating (serially diluted between 10^−1^ and 10^−6^) samples taken 0, 4, 23, 48, and 86 h post-antibiotic introduction on KB-agar and counting CFUs. Simultaneously, the subpopulations of resistant bacteria were counted by plating the same volume on KB-agar plates complemented with 200 μg/mL of streptomycin. Bacterial densities were estimated by averaging counts from two different samples. The population size detection threshold was 66 bacterial cells for the 0.2-mL populations, and 500 cells for the 1.5-mL populations (i.e., at least one CFU present in one of the 15-μL samples diluted to 10^−1^).

### Statistical analyses

Variation in population rescue probability was analyzed by means of logistic regression, with population survival (‘yes/no’) as a binary response variable. Explanatory factors included antibiotic dose and experimental treatment (‘clonal versus diversified’ population type in experiment 1; ‘large versus small volume’ population type in experiment 2). For hypothesis testing, deviances obtained from the logistic regression were used to calculate *F* values.

We further used repeated-measures analysis of variances to compare changes in (log-transformed) population size between future-rescued and extinct populations during the initial phase of the experiments (i.e., when all populations were still alive, but declining). In these analyses, time was log-transformed and taken as a covariate in the model. Population identity was added as a random factor to account for repeated measurements.

Where appropriate, we performed stepwise backwards model simplification, which involved the removal first of nonsignificant higher order interactions. All analyses were carried out using the JMP statistical package (SAS 2010).

## Results

### Experiment 1

**Figure 1:**
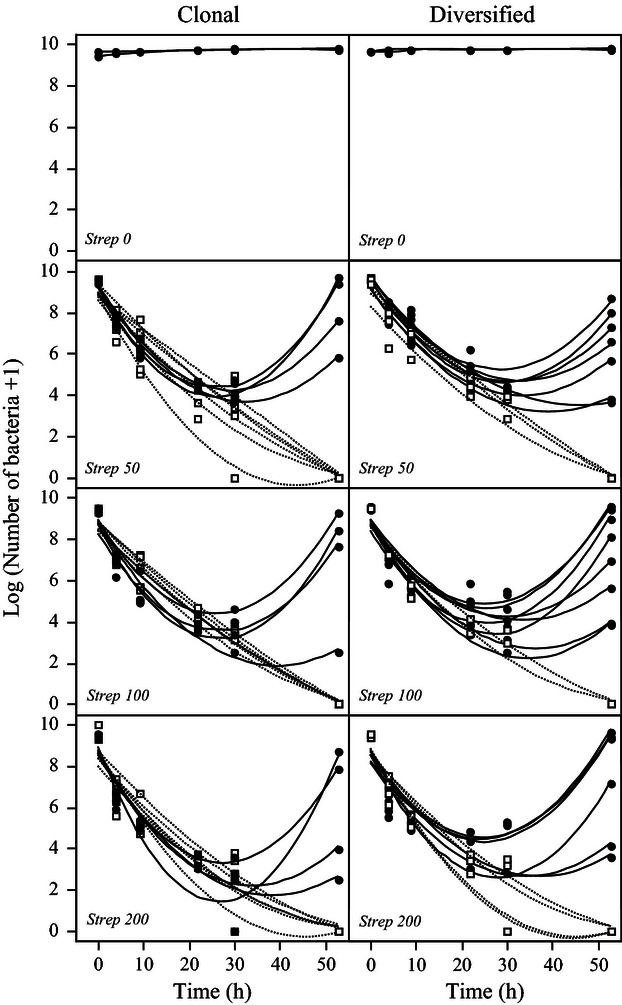
Population dynamics of rescued (solid lines and circles) and extinct (dashed lines and squares) bacterial populations by level of streptomycin (μg/mL) and by relative genetic diversity (clonal versus diversified) in experiment 1. Each line represents a single microcosm. Lines are nonlinear interpolations intended for illustration.

Exposure to lethal doses of streptomycin produced two distinct types of demographic dynamics. Either bacterial density continuously decreased until populations went extinct between hours 30 and 53, or populations declined for the first 20–30 h, but then grew again despite the presence of the antibiotic (Fig. 1). This latter response describes the U-shaped curve predicted for evolutionary rescues (Gomulkiewicz and Holt 1995; Bell and Gonzalez 2009). Population rescue was clearly associated with the evolution of antibiotic resistance: nearly all surviving populations (96.8%) were found to be resistant to streptomycin at the end of the experiment, whereas most of those populations going extinct (96.4%) were still sensitive to the antibiotic at the last time point before extinction.

**Figure 2:**
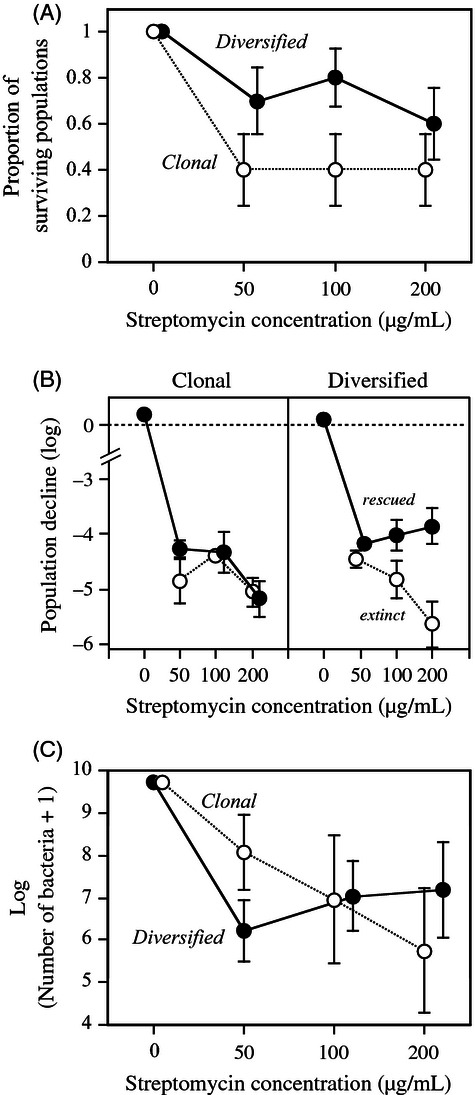
(A) Survival of clonal, low genetic-diversity populations and diversified, high genetic-diversity populations after 53 h in experiment 1, as a function of streptomycin concentration. (B) Mean population decline after 22 h (difference in log population size between 0 h and 22 h) of future-extinct and future-rescued populations as a function of population type and streptomycin concentration. (C) Mean population size (log-transformed) of rescued clonal and diversified populations after 53 h, as a function of streptomycin concentration. Error bars represent standard error (in (A) calculated from the binomial distribution; *n* = 10 populations).

Population type had a significant effect on the probability of evolutionary rescue (*F*_1,54_ = 5.13, *P* = 0.0276): 60–80% of the populations from the diversified treatment recovered from antibiotic stress, whereas only 40% of the populations from the clonal treatment did (Fig. 2A). Streptomycin concentration did not significantly affect population persistence (*F*_2,56_ = 0.21, *P* = 0.8112), nor was there a significant interaction with population type (*F*_2,54_ = 0.23, *P* = 0.7919).

Additional analysis (Table S1) revealed differences between diversified and clonal treatments during the initial 22 h, when all populations still had negative growth rates. Specifically, future rescued populations from the diversified treatment declined less rapidly than did those finally going extinct (significant treatment × rescue interaction: *F*_1,50_ = 4.61, *P* = 0.0366; Fig. 2B). Thus, evolutionary rescue in diversified populations could be detected before a net increase in population density was observable. Final density in rescued populations (53 h) varied considerably among individual replicates, and we found no significant overall difference between the diversified and clonal treatments (*F*_1,27_ = 0.03, *P* = 0.8750). However, all rescued populations still had lower densities than the antibiotic-free control populations (*F*_1,32_ = 5.62, *P* = 0.0237; Fig. 2C).

### Experiment 2

**Figure 3:**
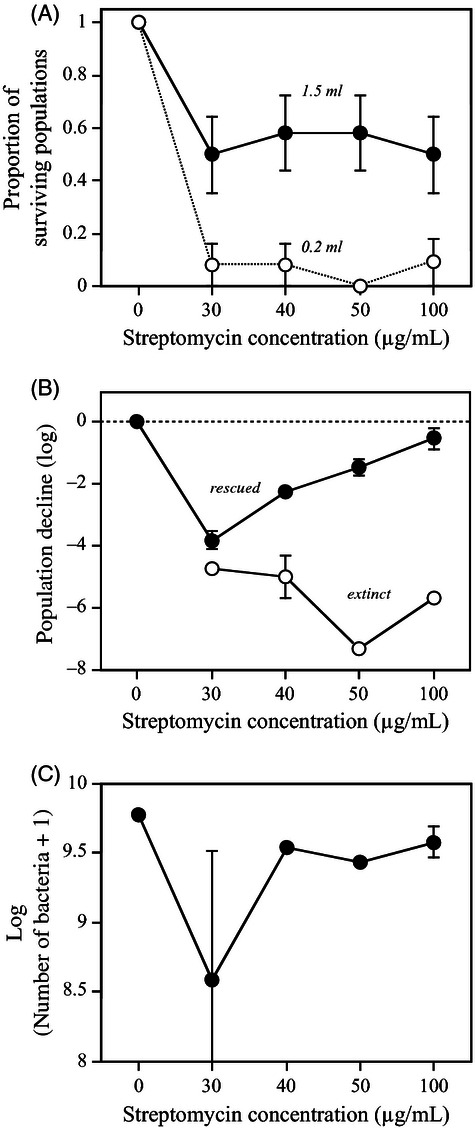
(A) Survival of small populations (0.2-mL microcosms) and large populations (1.5-mL microcosms) after 86 h in experiment 2 as a function of streptomycin concentration. (B) Mean population decline during day 2 (difference in log population size between 23 h and 48 h) of future-extinct and future-rescued populations in 1.5-mL microcosms, as a function of streptomycin concentration. (C) Mean population size (log-transformed) of rescued populations after 86 h in 1.5-mL microcosms, as a function of streptomycin concentration. Error bars represent standard error (in (A) calculated from the binomial distribution; *n* = 12 populations).

In this experiment, we tested for the effect of culture volume and therefore population size on the probability of rescue. Overall, 29 of the 95 bacterial populations (31%; one population lost due to handling error) survived antibiotic exposure, with little variation among the four doses (*F*_1,93_ = 0.19, *P* = 0.9030). Rescue curves had similar shapes to those observed in experiment 1 (Fig. S2). There was a significant effect of microcosm volume and thus initial population size on extinction probability of antibiotic-exposed populations (*F*_1,93_ = 29.81, *P* < 0.0001). Only 6.4% (3/47) of the small populations (0.2-mL volume) survived, whereas more than 50% (25/48) of the large populations (1.5-mL volume) showed evolutionary rescue from the antibiotic treatment after 86 h (Fig. 3A). There was no significant interaction between microcosm volume and antibiotic dose (*F*_1,87_ = 2.00, *P* = 0.1199).

**Figure 4:**
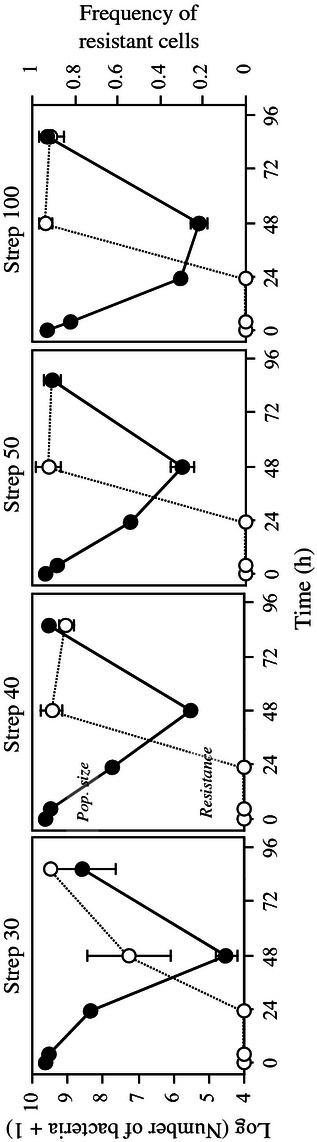
Rescue dynamics in large (1.5-mL) bacterial populations in experiment 2 as a function of streptomycin concentration. Changes in population size (left axis) are shown by solid circles. Changes in the frequency of antibiotic-resistant cells (right axis) are shown in open circles. Dynamics in nonrescued (extinct) populations are shown in Fig. S2; antibiotic-resistant cells were not observed in these populations. Error bars represent standard errors.

In all cases, population rescue was associated with a distinct antibiotic resistance mutant phenotype with detectable frequencies after 48 h (shown for 1.5-mL populations in Fig. 4). With the exception of one mutant CFU in one of the control populations at the last time point, no such resistance mutants were observed in control populations or in populations that did not survive antibiotic exposure.

We investigated in more detail the rescue dynamics in the 1.5-mL populations (Table S2). All antibiotic-exposed populations declined over the first 48 h of the experiment, and for future extinct populations, stronger declines were associated with higher streptomycin doses (Fig. 3B). In contrast, during day 2 of the experiment, declines were less steep in the future-rescued populations (significant effect of rescue: *F*_1,40_ = 1210, *P* < 0.0001, Table S2; Fig. 3B), in particular at higher streptomycin doses (significant rescue × dose interaction: *F*_3,40_ = 11.20, *P* < 0.0001, Table S2; Fig. 3B).

Furthermore, the reduced population decline of future rescued populations was associated with the concomitant appearance of resistant mutants. For all populations combined, the density change between 24 and 48 h was positively correlated with the frequency of resistant mutants at 48 h (*r* = 0.46, *n* = 26, *P* = 0.0182; Fig. S3). That is, populations with a relatively small decline in density (or even an increase) harbored higher frequencies of resistant mutants. Thus, as in experiment 1, a signature of evolutionary rescue was detectable before population density actually began to increase, and this signature was correlated with the increasing frequency of resistant mutants.

At the end of the experiment (86 h), there were no significant effects of antibiotic dose on resistance mutant frequency or population density (*P* > 0.3); and final density tended to be lower in rescued than in control populations, although this difference was not statistically significant (*F*_1,33_ = 2.40, *P* = 0.1308; Fig. 3C).

## Discussion

Our results show how initial genetic diversity and population size promote the chances that evolution will save a bacterium population from extinction when confronted with an otherwise lethal antibiotic. We observed that such ‘evolutionary rescues’ followed the U-shaped trajectories predicted by theoretical models (Gomulkiewicz and Holt 1995; Orr and Unckless 2008). Specifically, we confirm previous theoretical (de Visser and Rozen 2005; Handel and Rozen 2009) and experimental (Bell and Gonzalez 2009) results that larger populations have a higher probability of evolutionary rescue. Population size may influence evolutionary rescue through two distinct mechanisms. First, the time available for a population to ‘find’ an evolutionary solution depends on initial density; that is, the ability of a population threatened by extinction to demographically sustain itself (Gomulkiewicz and Holt 1995). Second, at a constant per capita mutation rate, a larger population will have higher standing genetic variation and hence greater adaptive (and rescue) potential (Willi et al. 2006; Kawecki 2008). Here, we disentangled the impacts of population size and standing variation by directly manipulating initial genetic diversity, without changing population size. The higher proportion of rescued populations in the diversified treatments clearly highlights the important role of genetic diversity in providing rescue mutants. Although we were not able to determine the actual mechanism leading to rescues and explaining the differences between treatments in Fig. 2B, our data showed that there was, on average, more than an order of magnitude greater number of resistant mutants in the diversified compared with the clonal populations (left panel, Fig. S1). A parsimonious explanation for the maintenance of these potentially costly mutants prior to antibiotic exposure is a mutation-selection balance.

### Evidence for genetically based antibiotic resistance

Several lines of evidence support the hypothesis that the rescues were due to the spread of heritable resistance and not to phenotypic tolerance. First, we demonstrated that resistance to streptomycin is heritable by culturing bacteria from rescued populations in fresh antibiotic-free conditions for 24 h (*c*. seven generations) before retesting their resistance against streptomycin. Had the rescues been due solely to phenotypic tolerance, the supposedly tolerant cells would have reverted to sensitivity in the benign environment. They did not, indicating that resistance to streptomycin in our rescued populations is a genetically heritable trait.

Second, nonheritable resistance to antibiotics has been reported in another pseudomonad (*P. aeruginosa*) against streptomycin (Xiong et al. 1997; see also Skiada et al. 2011). However, this phenotypic resistance did not last for more than 24 h (Xiong et al. 1997), and (persister) cells that could be responsible for such resistance are either not known in *P. fluorescens* SBW25 or involve biofilm structures (Levin and Rozen 2006). Biofilms can form on the inner walls of microcosms (MacLean et al. 2005), and we cannot totally exclude their role in observed rescues, for instance, if they provided phenotypic resistance that subsequently permitted the emergence of *de novo* genetically resistant mutants. However, the most parsimonious explanation for the genetically based resistance we observed in rescued populations at the end of the experiments is the presence of genetically based resistant mutants at the beginning of the experiments (Fig. S1). Finally, in experiment 2, the appearance of the resistant phenotype was exclusively restricted to populations undergoing rescue. Had there been a physiologically based, plastic reaction to the antibiotic, some level of this resistance phenotype should have been detectable in the nonrescued populations.

And third, we verified that streptomycin was still present in sufficient concentrations to kill susceptible cells after 72 h of incubation with bacteria (see Supporting information, Assay 4 in Data S1). Thus, only persister cells, biofilm structures, or specific genetically resistant cells could explain the rescues observed, and as argued above, the former two explanations are unlikely in our experimental system.

### When did the resistant mutants appear?

Under the simplifying assumption that the observed rescues were due to a single point mutation, then we could theoretically estimate the time of mutant appearance (i.e. before or after introduction of the antibiotic) by extrapolating the line of exponential regrowth back in time. However, in addition to assuming that only a point mutation is required and that the rescue is due to a single cell, this method ignores delays in the onset of exponential growth of the mutant and demographic stochasticity (see Martin et al. 2013).

There are several reasons for why the rescues we observed could be consistent with a single streptomycin-resistant mutant genotype being present before the antibiotic was actually introduced. First, we estimated the spontaneous (i.e. without antibiotic pressure) mutation rate from susceptibility to streptomycin resistance to be *c*. 1 cell in every 3.94 × 10^9^ (see Supporting information, Assay 2 in Data S1). Assuming the maximal densities in our microcosms to be 2–5 × 10^9^ cell/mL (Escobar-Paramo et al. 2012), this suggests it is unlikely that small populations (0.2 mL) would harbor spontaneous resistant mutants before antibiotic introduction. This is consistent with the very low rate of rescue in 0.2-mL populations and the *c*. 50% probability in 1.5-mL populations in experiment 2, although we cannot exclude that *de novo* mutations contributed to rescues.

Second, for an initial single resistant cell to attain a carrying capacity of *c*. 2 × 10^9^ cells, 31 doublings would be necessary, and based on our estimate of a maximum rate of *c*. 40 min per doubling (see Supporting information, Assay 5 in Data S1), this would require about 21 h. This estimate assumes uninterrupted exponential growth, but nevertheless it is well under the 53 h of the first experiment, meaning that even in adding an initial lag phase and final competition (i.e. stationary phase) to the growth estimate, it would be consistent with a small number of initial mutants causing the rescue.

Thus, our results indicate that one or a small number of mutants already present in the population is sufficient to explain the rescued populations that returned to high densities. Yet, even though almost all rescued populations showed this behavior in experiment 2 (Fig. S2), important but transient differences in densities were observed between populations during the rescue process (Fig. 1 at 53 h; Fig. S2 at 48 h). In populations where densities decreased to the lowest levels before growing again, associated late rescues could have been either the result of the appearance of a *de novo* mutation or a long delay before exponential growth of a pre-existing mutant due to demographic stochasticity (Martin et al. 2013). Indeed, certain antibiotics are known to be mutagenic (e.g., Kohanski et al. 2010), but we do not know whether this mechanism played a role in the patterns observed in this study.

### Pharmacological implications

Our experiments are comparable to standard tests in pharmacological research used to determine effective antibiotic treatment protocols (‘time-kill experiments’). Our results are in line with common findings of these tests, namely rapid population decline following antibiotic exposure and the subsequent slowing down of kill rates (thus resulting in U-shaped density curves). In the pharmacological literature, the ‘Inoculation effect’ is also well known: antibiotic treatments become less efficient at higher initial bacterial densities (Udekwu et al. 2009). However, unlike our study, these attenuated effects of antibiotics are often not attributed to the appearance of heritable, genetically based resistance mutants (Regoes et al. 2004; Udekwu et al. 2009; Levin and Udekwu 2010; Skiada et al. 2011). In part, this discrepancy illustrates that different organisms and/or antibiotics can produce different outcomes. It is also possible that we were able to detect genetically based antibiotic resistance because we employed relatively long time windows (up to 4 days) and relatively large populations. This suggests that including genetic adaptation will be important at least for predicting pharmacodynamics of large infection foci treated over longer time spans, such as chronic infections of *P. aeruginosa*, a close relative of *P. fluorescens*.

## Conclusion

Our experiments demonstrate that initial population size and genetic diversity play important roles in the probability of evolutionary rescue. They also suggest that early population dynamics may predict to some extent future evolutionary rescue: rescued populations in the diversified treatment initially declined less rapidly than did populations finally going extinct. This finding could prove useful for assessing the extinction risk in natural populations.

## Data archiving statement

Data deposited in the Dryad repository: doi:10.5061/dryad.c59q3.

## Supporting Information

**Figure S1.** Estimated range of initial number of resistant cells in each vial or well of the different treatments in experiments 1 and 2 as a function of population types (clonal or diversified) and culture volumes. The number of resistant cells is calculated using the initial density of resistant cells measured in clonal populations (1 out of every 3.94 × 10^9^cells, see supplementary assay 2 in Data S1) and diversified populations (1 out of every 2.285 × 10^8^ cells, see supplementary assay 1 in Data S1). In experiment 2, an initial number of resistant cells below 1 (dashed line) corresponds to the probability of each well harbouring a single resistant cell at the beginning of experiment.

**Figure S2.** Rescue dynamics in experiment 2 shown for small (0.2 mL) and large (1.5 mL) populations and for different streptomycin concentrations (µg/mL). Each line represents a single microcosm population. Future rescued populations shown with solid circles, future extinct populations with open circles. Lines are non-linear interpolation intended for illustration.

**Figure S3.** Correlation between the rate of population decrease and the increase of resistance combined over all future rescued 1.5 mL replicate populations in experiment 2. Population decrease is calculated as the difference in log-transformed population size between 23 and 48 h of streptomycin exposure. The frequency of resistant cells is taken at *t* = 48 h (no resistance was detected at *t* = 23 h). Overall, a slower population decline was associated with a higher frequency of resistant cells, indicating a direct link between population dynamics and selection for antibiotic resistance. A regression line illustrates this relationship.

**Figure S4.** Population size dynamics of naive *P. fluorescens* exposed to 72-h old media. Old media had been started either with (solid lines) or without (dashed lines), streptomycin, and had contained bacteria (square symbols) or not (round symbols). All media replicates were filtered before adding samples of naive bacteria. Media initially with or without bacteria and with antibiotics still trigger population decrease after 72 h. Means and standard errors based on 3 replicates.

**Table S1.** Repeated-measures Analysis of Variance of (log-transformed) population size in experiment 1. Population type (clonal versus diversified), streptomycin dose (50, 100, 200 µg/mL) and population rescue (extinct versus alive at *t* = 53 h) are explanatory factors. Analysis restricted to the first 22 h of antibiotic exposure, during which time all populations declined in density. Time (+1 h) was log-transformed and taken as a covariate in the model. Population identity was added as a random factor to account for repeated measurements.

**Table S2.** Repeated-measures Analysis of Variance of (log-transformed) population size during the first 48 h of experiment for the 1.5 mL treatment. Streptomycin dose (30, 40, 50, 100 µg/mL) and population rescue (extinct versus alive at *t* = 86 h) were taken as explanatory factors. Time (+1 h) was log-transformed and taken as a covariate. Population identity was added as a random factor to account for repeated measurements.

**Data S1.** Assay Methods and Results.
