# Supplementary material for: Evolutionary rescue in populations of Pseudomonas fluorescens across an antibiotic gradient
Source: Evol Appl. 2013 Feb 4;6(4):608–16. doi: 10.1111/eva.12046 (PMC3684742; doi:10.1111/eva.12046)
Supplement: Supplementary file 1 [file eva0006-0608-SD1.pdf]

Evolutionary rescue in populations of *Pseudomonas fluorescens* across an antibiotic gradient

Johan Ramsayer[1], Oliver Kaltz[1#] and Michael E. Hochberg[1,2#]

[1] Institute of Evolutionary Sciences - Montpellier (UMR 5554 ISE-M), University of Montpellier 2, France

[2] Santa Fe Institute, 1399 Hyde Park Road, Santa Fe, NM 87501 USA

Supplementary information:

Assay methods and results

Figures S1-S4

Tables S1 – S2

Supporting information: Assay methods and results

Assay 1. Estimation of the initial resistant mutant density in the diversified “master” population.

The initial density of resistant mutants in the diversified “master” population was measured after the experiments using an a priori identical master population (see materials and methods section of first experiment). Five replicate samples were created from the master population, 20 µl of each sample was plated undiluted onto KB-agar plates complemented with 200 µg/ml streptomycin (to count the number of resistant colonies), and 20 µl of each sample was diluted to 10^{-6} of their initial density and plated on standard KB-agar plates (to measure total density). All bacteria were grown for 48 hours at 28 °C before counting.

The number of resistant cells is estimated to be 1 out of every 2.285x 10^{8}. Very few colonies (between 6 and 9) were able to grow on any of the plates containing streptomycin.

Assay 2. Estimation of spontaneous mutation rate from sensitivity to resistance. During experiment 2, we also assayed the 24 control populations (i.e. without antibiotics) by plating them on KB-agar complemented with 200 µg/ml of streptomycin to test for the spontaneous appearance of resistant mutants.

The number of cells resistant to streptomycin was *c.* 1 out of every 3.94 x10^{9} cells. This corresponds to the initial frequency of resistant mutants in the non-diversified populations. 96% of the replicates did not show a single resistant colony.

Assay 3. Estimation of the MIC_{50} for Streptomycin on *Pseudomonas fluorescens* SBW25. We measured the MIC_{50} (i.e. the concentration of streptomycin that reduces bacterial population size after a specified time by half) in a 96 well plate, by inoculating 5 µl of *P. fluorescens* SBW25 in 100 µl of KB medium complemented with decreasing doses of streptomycin (150, 75, 37.5,

18.75, 9.375, 4.688, 2.344, 1.172, 0.586, 0.293 and 0 µg/ml). Each dose treatment was replicated three times. Three replicated wells of KB alone served as controls. The populations grew for 24 hours at 28 °C under constant agitation (130 rpm) (the approximate time for control populations to attain maximum densities), at which time all population densities were measured with a spectrophotometer (600 nm).

After 24 hours, bacterial density was 39.8% lower than controls at a dose of 18.75µg/ml and 89.7% lower at 37.5µg/ml. By linear interpolation we estimate the concentration of streptomycin that reduces the bacterial growth by half (= MIC_{50}) to be *c.* 22.6 ± 2.04 µg/ml.

Assay 4. Streptomycin activity after 72 h. To evaluate whether population recovery was the result of adaptation and not due to reduced antibiotic activity, we tested whether streptomycin was still present and active after 72 h of incubation with bacteria. We started four types of cultures: one treatment with 2ml of *P. fluorescens* at carrying capacity in KB medium plus streptomycin at 100 µg/ml, and three controls: 2 ml of KB medium only, 2 ml of KB and 100 µg/ml of streptomycin, and 2 ml of *P. fluorescens* at carrying capacity in KB. Microcosms were incubated at 28 °C under constant orbital agitation for 72 h, and then centrifuged for 10 min at 8000 rpm. To evaluate the impacts of the resulting aliquots on bacterial growth, we centrifuged 200 µl samples of ancestral *P. fluorescens* after 24 hours of growth in KB. The liquid medium was removed and replaced with sampled media (3 replicates for each type of medium). We measured resulting population densities by diluting and plating 20 µl samples on KB-agar plates at T = 0, T = 75 min and T = 275 min post exposure to the treatment and three controls described above.

The streptomycin aliquots decreased population density after 72 h of incubation alone or with bacteria ($F_{1,10}$ = 26.48, p = 0.0009) (Fig. S4). This indicates that the antibiotic imposed selection pressure for bacterial resistance over the entire period of the main experiments.

Assay 5. Estimation of minimum doubling time. We measured the minimum doubling time of streptomycin-resistant *P. fluorescens* in two environments: KB and KB supplemented with 50 µg/ml of streptomycin. We also estimated the doubling time of the ancestor in KB. Cultures were started at 10% of final bacterial density in 200 µl of KB in a 96 well plate (12 replicates for each treatment). We incubated plates for 7 hours at 28 °C until they reached the exponential growth phase and then measured their population densities at T = 7 h and 8 h by plating 20 µl samples on KB-agar plates. Doubling time was calculated using the formula: $DT = (T_2 - T_1) * (Log(2) / Log(D_{2\,-}\,D_1))$.

We found the minimal doubling time of the streptomycin-resistant strain of *P. fluorescens* to be 40.7 ± 8.3 minutes in the presence of streptomycin and 48.2 ± 15.94 without streptomycin. These values were not significantly different from the doubling time of the sensitive strain (41.2 ± 7.53 minutes; $F_{2,21} = 0.4022$; $p = 0.6722$).

Figure S1. Estimated range of initial number of resistant cells in each vial or well of the different treatments in experiments 1 and 2 as a function of population types (clonal or diversified) and culture volumes. The number of resistant cells is calculated using the initial density of resistant cells measured in clonal populations (1 out of every 3.94 x10^9 cells, see supplementary assay 2 in Data S1) and diversified populations (1 out of every 2.285 x10^8 cells, see supplementary assay 1 in Data S1). In experiment 2, an initial number of resistant cells below 1 (dashed line) corresponds to the probability of each well harbouring a single resistant cell at the beginning of experiment.

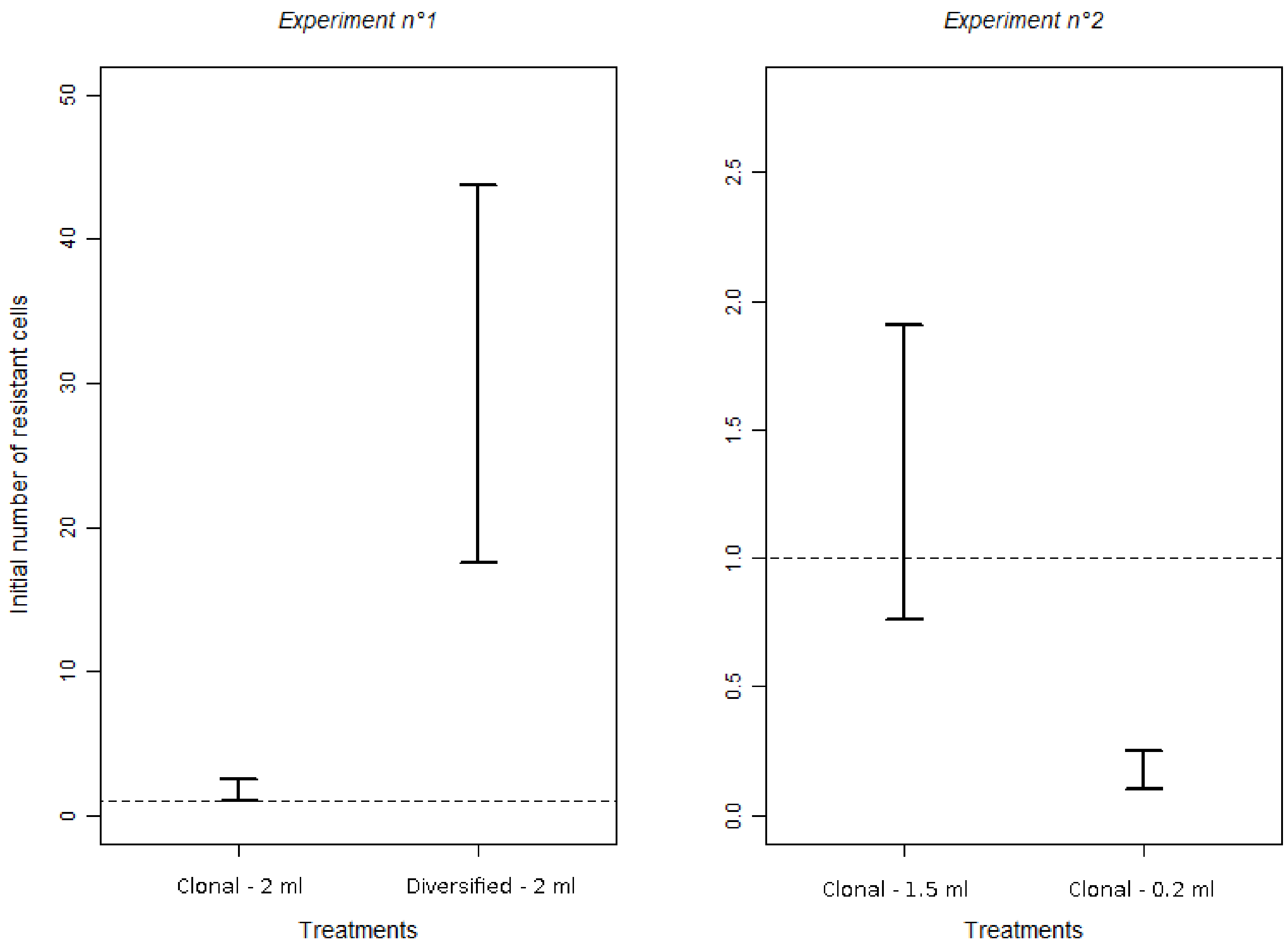

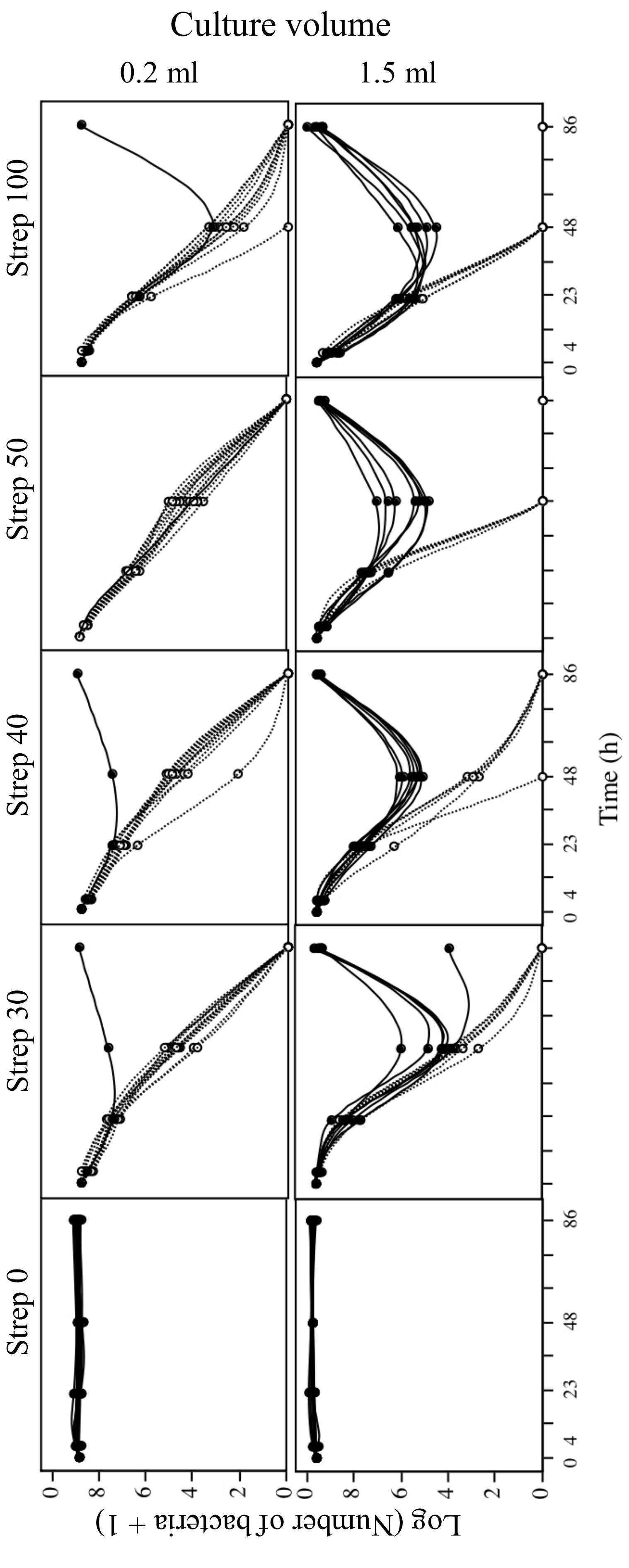

Figure S2. Rescue dynamics in experiment 2 shown for small (0.2 ml) and large (1.5 ml) populations and for different streptomycin concentrations (μg/ml). Each line represents a single microcosm population. Future rescued populations shown with solid circles, future extinct populations with open circles. Lines are non-linear interpolation intended for illustration.

Figure S3: Correlation between the rate of population decrease and the increase of resistance combined over all future rescued 1.5 ml replicate populations in experiment 2. Population decrease is calculated as the difference in log-transformed population size between 23 and 48 h of streptomycin exposure. The frequency of resistant cells is taken at t = 48 h (no resistance was detected at t = 23 h). Overall, a slower population decline was associated with a higher frequency of resistant cells, indicating a direct link between population dynamics and selection for antibiotic resistance. A regression line illustrates this relationship.

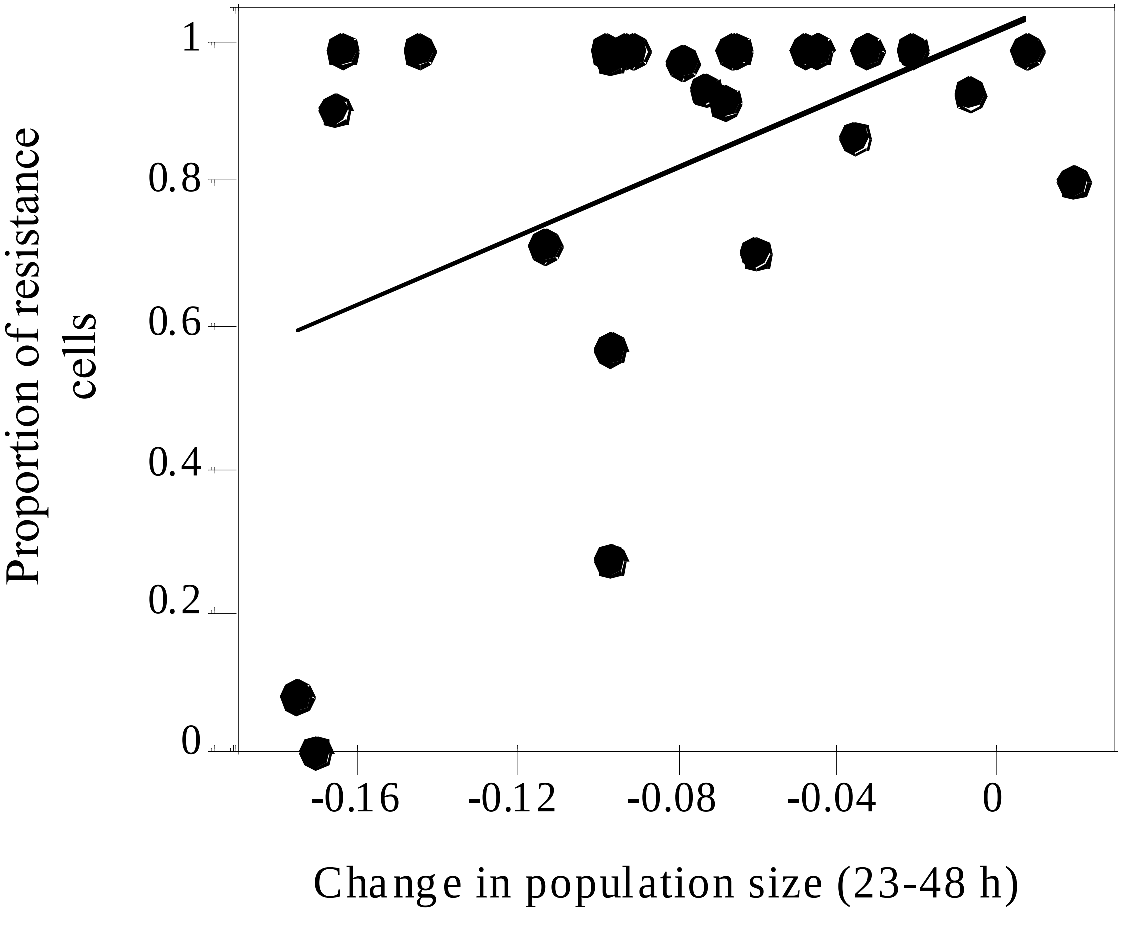

Figure S4. Population size dynamics of naive *P. fluorescens* exposed to 72-h old media. Old media had been started either with (solid lines) or without (dashed lines) streptomycin, and had contained bacteria (square symbols) or not (round symbols). All media replicates were filtered before adding samples of naive bacteria. Media initially with or without bacteria and with antibiotics still trigger population decrease after 72h. Means and standard error based on 3 replicates.

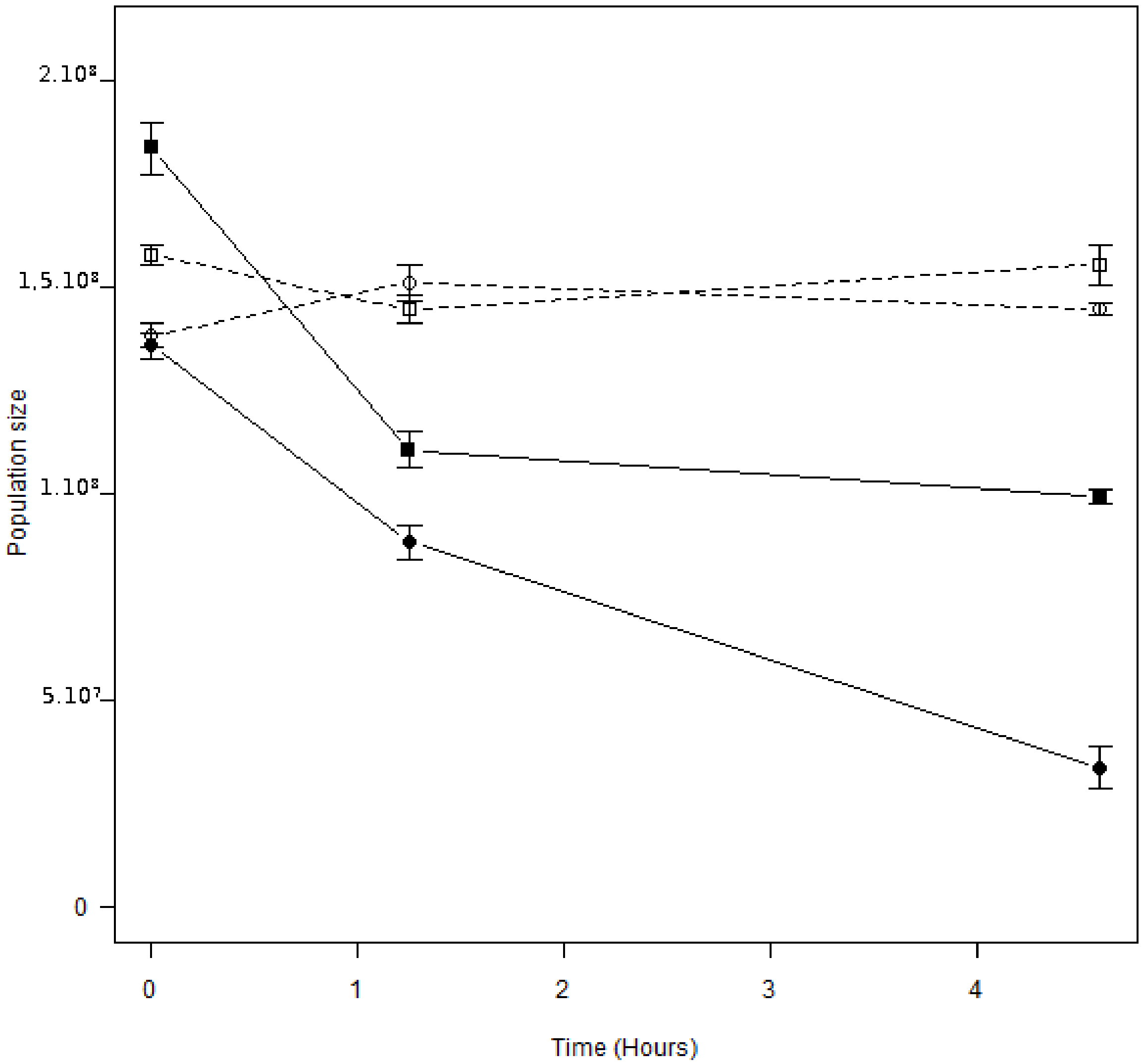

Table S1. Repeated-measures Analysis of Variance of (log-transformed) population size in experiment 1. Population type (clonal vs. diversified), streptomycin dose (50, 100, 200 μg/ml) and population rescue (extinct vs. alive at t = 53 h) are explanatory factors. Analysis restricted to the first 22h of antibiotic exposure, during which time all populations declined in density. Time (+1h) was log-transformed and taken as a covariate in the model. Population identity was added as a random factor to account for repeated measurements.

Source (fixed effects)	df, denom. df	F	P
Population type	1, 50	3.60	0.0635
Streptomycin dose	2, 50	35.10	0.0001
Population rescue	1, 50	0.06	0.8115
Pop type x Rescue	1, 50	4.61	0.0366
Pop type x Dose	2, 50	1.44	0.2458
Dose x Rescue	2, 50	3.12	0.0530
Time	1, 52	5125.54	0.0001
Pop type x Time	1, 52	2.77	0.1018
Dose x Time	2, 52	18.67	0.0001
Rescue x Time	1, 52	2.10	0.1536
Pop type x Rescue x Time	1, 52	6.92	0.0112
Pop type x Dose x Time	2, 52	4.42	0.0169

Table S2. Repeated-measures Analysis of Variance of (log-transformed) population size during the first 48h of experiment 2 for the 1.5 ml treatment. Streptomycin dose (30, 40, 50, 100 µg/ml) and population rescue (extinct vs. alive at t= 86h) were taken as explanatory factors. Time (+1h) was log-transformed and taken as a covariate. Population identity was added as a random factor to account for repeated measurements.

Source (fixed effects)	d.f., denom. d.f.	F	P
Streptomycin dose	3, 40	22.65	< 0.0001
Population rescue	1, 40	1210.41	< 0.0001
Dose x rescue	3, 40	11.20	< 0.0001
Time	1, 184	292.09	< 0.0001
Dose x time	3, 184	0.21	0.8923
Rescue x time	1, 184	108.48	< 0.0001
Dose x rescue x time	3, 184	0.72	0.5421
